# Construction of neural network model for exercise load monitoring based on yoga training data and rehabilitation therapy

**DOI:** 10.1016/j.heliyon.2024.e32679

**Published:** 2024-06-07

**Authors:** Wenhui Ma, Bin Guo

**Affiliations:** a College of Physical Education, China Three Gorges University, Yichang, 443002, Hubei, China; b Graduate School, Philippine Christian University, Malate, Manila, 1004, Philippines; c School of Physical Education, DaLian University, DaLian, 116622, Liaoning, China

**Keywords:** Sports load detection, Yoga training, Rehabilitation and treatment, Neural network model construction of neural network models

## Abstract

The Internet of Things is based on the traditional Internet and its purpose is to achieve information exchange between users and devices, as well as between devices. The rapid development of sensor technology, communication network technology, and computer technology has enriched the coverage of the Internet of Things, including a wide range of intelligent applications such as healthcare, smart cities, and smart homes. The development of high-performance computing and machine learning technologies has promoted the wide application of intelligent auxiliary systems in sports medicine. With the rapid development of yoga in the field of sports, athletes can play the various functions of yoga, improve their physical strength and quality, and improve their strength, flexibility, etc., cultivate positive, optimistic, and healthy emotions, and these are conducive to rehabilitation treatment after sports injuries. Therefore, it is feasible and feasible to introduce yoga training into the monitoring of the exercise load of athletes. In this paper, neural network technology was used to break the traditional training method based on experience. Based on yoga training data, through experimental exercise research, it could explore a new effective way to monitor exercise load and rehabilitation treatment, and build an exercise load monitoring model of the Ant Colony Optimization (ACO) neural network. By sorting out the data, statistics and analysis of the data, this article confirmed the effect of yoga training on reducing fatigue after exercise. The experimental results showed that the prediction value obtained by the ACO neural network model was 9.106, and the error was only −0.003 compared to the actual detection value of 9.109. This result showed that the ACO neural network model can perfectly fit the functional relationship between yoga training level and exercise load and has high prediction accuracy. This also marked that the development of high-performance computing systems has entered a new journey in the field of sports and health.

## 1 Introduction

Data mining, also known as knowledge discovery, aims to extract information from data sets using intelligent methods [1]. The task of data mining is to perform semi-automatic or automatic analysis and processing on a large amount of data, extract hidden and valuable potential information from disorderly data, and apply the results of data analysis to practical applications [2,3]. The introduction of machine learning technology into the field of sports medicine is conducive to the realization of intelligent diagnosis of athletes. The results of the research and the detection methods in sports physiology and biochemistry have promoted the development of sports scientific research in practical application. At present, athletes' sports load monitoring technology has been relatively mature. Many monitoring indicators and methods are simple and easy to achieve, and can be detected in real time according to different items, so as to grasp the load status. In exercise load monitoring, it can also refer to some physiological indicators that can better evaluate and analyze the athlete's exercise load to ensure that athletes can get good training results. Scientific researchers use various indicators to monitor athletes' exercise load, analyze, and evaluate athletes' training effect and training quality, which is an important condition for implementing scientific training, and it is also the most widely used item in sports training.

However, the quantitative analysis of the combination of the training methods of athletes and the exercise load is very complex, and there is not much research on this aspect currently. Iolascon Giovanni pointed out that many coaches lack scientific and quantitative statistical analysis and monitoring management when making training plans and programs for athletes, which were not conducive to the organization of follow-up sports plans [4]. Andrews and Sophie C emphasized that the training pressure under which athletes are under should be a comprehensive evaluation of multiple indicators, levels, and factors, and the athlete's exercise load is a very critical factor [5]. Miranda-Comas Gerardo believed that the analysis of the characteristics of sport load aimed mainly at changing the characteristics of load and intensity produced by athletes in different training stages, and most studies focus mainly on pre-contest training [6]. Bauer Pascal's study found that the effect of exercise became worse under high load. Therefore, it was very necessary to monitor the exercise load of athletes on the premise of ensuring that the athletes' bodies are fully stimulated and preventing sports injuries [7]. Imbach and Frank showed that the use of a neural network to optimize training and establish the corresponding relationship with exercise load is conducive to a comprehensive analysis of athletes [8]. According to the above analysis, it can be seen that scientific and reasonable adjustment of athletes' sports load is the key to improving the competitive level.

Yoga is a new static exercise method. Shaw emphasized that yoga was a truly physical and mental sport, which was different from other sports. It can not only improve the physical fitness of athletes, but also promote their physical and mental health, and attach importance to personal experience [9]. Mir Fatemeh explored the effects of different content, forms, and physiological loads of yoga and aerobic exercise on improving athlete physical fitness, and the results showed that long-term yoga practice was effective in improving athlete physical conditions [10]. Richmond Diane said that although yoga does not play an important role in improving the metabolism of athletes, it played a significant role in developing flexibility, strength, and reducing physical fatigue [11]. Phuhanich Melissa E pointed out that yoga exercise can help stretch the muscles of the body, increase the range of motion of the joints, and promote the flexibility and endurance of all parts of the body [12]. In summary, yoga has a small load, which is conducive to regulating the physical condition and psychological pressure of athletes. Therefore, this study is of very important significance and useful value.

Yoga pays attention to self-culturing, both internal and external. It does not require too much physical strength and the equipment required is simple, so young people will welcome and love it. However, in theory or practice, yoga teaching is in the process of exploring reform and optimization. Whether fitness programs like yoga can meet the requirements of sports activities, there is still a wide debate. In general, it is believed that during exercise the heart rate of the physiological load should be between 120 and 140. The core of healthy sports is that sports should have an appropriate amount of exercise. It is recommended that the intensity of each exercise be between 140 and 160 times/minute. The average heart rate of yoga training does not exceed 120 times. Obviously, if yoga is the main training item, it is difficult to achieve this requirement. However, the actual investigation and research shows that practitioners often feel “very tired” after practice, which conflicts with the low heart rate in yoga training. Therefore, scientific monitoring methods are used to objectively evaluate the fitness effect of yoga, find its fitness value, and point out its shortcomings, in order to provide a theoretical basis for the development of yoga and other health fitness activities.

The goal of this paper is to establish an exercise load monitoring model based on a neural network algorithm. By integrating the ACO neural network and yoga training data, the objective is to provide coaches and athletes with a reliable tool to scientifically evaluate and monitor exercise load, optimize training plans, improve sports performance, and reduce the risk of sports injuries.

The contribution of the study of building an exercise load monitoring model of an ant colony optimization (ACO) neural network is mostly evident in the following elements, which are based on data from yoga training and rehabilitation treatment: 1. The accuracy of exercise load monitoring has increased. The accuracy of previous exercise load monitoring techniques was limited and frequently depended on experience or basic statistical models. Exercise load can be anticipated and tracked more precisely with the introduction of the ant colony optimization (ACO) neural network model, and yoga training and rehabilitation treatments can benefit from more reliable and scientific data support.2. Optimal rehabilitation treatment plan: A crucial component of rehabilitation care is monitoring exercise load. Rehabilitation therapists can provide patients with more detailed and in-depth information about their ability to exercise by correctly assessing the patient's activity load. This improves the efficacy and efficiency of rehabilitation treatment by allowing the creation of more precise and individualized treatment regimens.

## 2 Yoga training and physical rehabilitation therapy

### 2.1 Features of Yoga training

With the promotion of the concept of scientific exercise, the physical health of athletes has received increasing attention. The monitoring of exercise load has become an important way for coaches to pay attention to the health of athletes, and yoga is conducive to relieving physical fatigue from athletes. In the face of chaotic data, this article uses machine learning to perform information processing on yoga-based training data, uses the processed data for model training after excessive performance calculation, and finally obtains the actual results of exercise load monitoring [13].

**Fig. 1:**
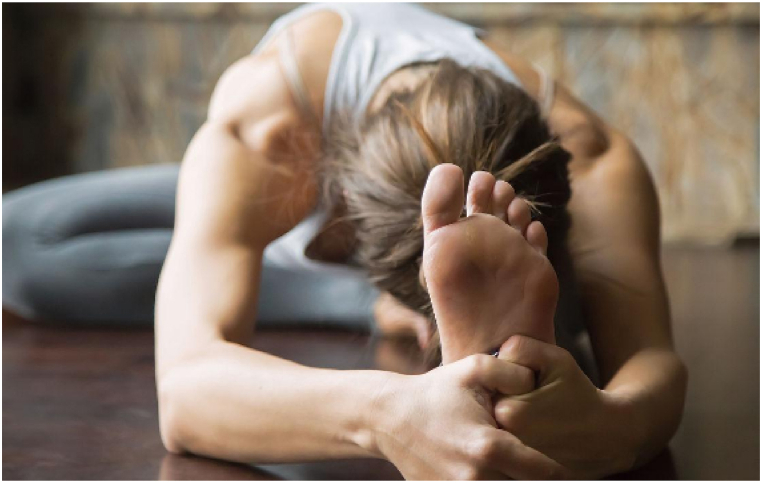
Examples of yoga exercises.

Yoga means integration, connection, and unification. It is an art, a science, and an attitude towards life. During exercise, people should concentrate fully and relax their bodies so that they can have a full rest. Yoga is not traditional hypnosis [14,15]. This kind of rest is fundamentally different from ordinary sleep. They are two completely different areas. Although your starting point is to relax and accept, your ultimate goals are different. In yoga, when the trainer can cut off perception and maintain perception, he can cross personality barriers and enter a new or deeper level, and consciousness would bring the trainer to the limit of guidance, which is the goal of yoga. The example of yoga training actions is shown in Fig. 1.

**Fig. 2:**
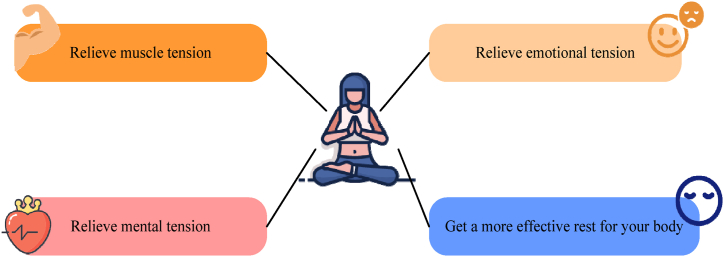
Features of yoga.

As can be seen in Fig. 1, during training, the trainer is in a relaxed state without distractions, but the trainer should also pay attention not to doze off and try to stay awake. In terms of perception, the trainer knows what he is doing, because his mind is very clear when practicing yoga. The trainer's brain would still operate freely and experience a different kind of consciousness. During hypnosis, the hypnotized person would enter a deep sleep state. At this time, his brain is completely closed and his thinking would be limited to a narrow range. The human body would become lazy. Therefore, yoga can help people who are tired and lack sleep quickly enter a relaxed state, replenish their physical strength, and improve sleep quality [16,17]. The characteristics of yoga are shown in Fig. 2.

As shown in Fig. 2, yoga can relieve muscle tension, which is mainly caused by the imbalance of the human body, the nervous system, and the endocrine system. By practicing yoga, athletes can completely relax their body and mind and ultimately eliminate this imbalance in the body. Yoga can relieve emotions. Emotional tension is caused by various dualistic differentiations, such as love, hate, gain and loss, success and failure, happiness and sadness, etc. These are difficult to overcome because people cannot express their feelings freely. This kind of pressure cannot be eliminated by sleeping and relaxing, because people are disgusted with it from the bottom of their hearts, but yoga can make their hearts peaceful. Yoga can reduce psychological pressure. Excessive psychological activities, including crankiness and indecision, can cause psychological pressure. This is a kind of mental pressure, because memory would be stored in consciousness, thus affecting the physical and mental state. Yoga can give the body better rest. Yoga is a scientific relaxation method, which can release the emotions of athletes in an unconscious state and keep the body in harmony. If athletes want to have better rest, the first thing they need to do is relax. Relaxation is relatively easy as long as you lie down and keep your eyes closed. After being squeaky, the athletes would feel a temporary happiness. In fact, their spirit and body are not completely relaxed but rather a kind of pressure. Scientifically speaking, yoga plays a much better role in sports training of athletes than in normal sleep [18]. People who use this method every day would find that their sleep habits have changed dramatically in a short period of time. For athletes, yoga training can fully relax the body and spirit, and its actual effect is better than traditional routine sleep.

The characteristics of yoga are mainly because it includes a lot of stretching, breathing, movement, balance, meditation, and strength. Compared to doing boring exercises in the gym, yoga is much more interesting. Those who often do yoga often feel that yoga would become more interesting as time goes on. The effect of yoga is lasting. First of all, yoga can relax the body and spirit and relieve pain in a short time [19]. Second, with continuous exercise, especially posture training and adjustment of the breathing method aimed at stretching and strengthening the body, the body and breathing ability can be greatly improved. Finally, with improvement in body and lung functions, intestinal function and the immune system would also be improved. In fact, yoga is a way to exercise balance. Many people believe that yoga is mainly exercise for the flexibility of the body. However, this is not the main purpose of yoga; it is to maintain the balance of the body as the main goal. In the opinion of many people, yoga mainly exercises the flexibility of the body. However, this is not the primary goal of asana yoga, it is balance. Some yoga practitioners, especially some women, are originally very flexible, but lack strength. Others, including some men, are very strong but lack flexibility. Some yoga students become cowards because of fear, some are often listless, and some cannot completely relax. In any case, what yoga should do is challenge yourself, turn obligations into motivation, and become a balanced person.

### 2.2 Sports load and rehabilitation treatment

**Fig. 3:**
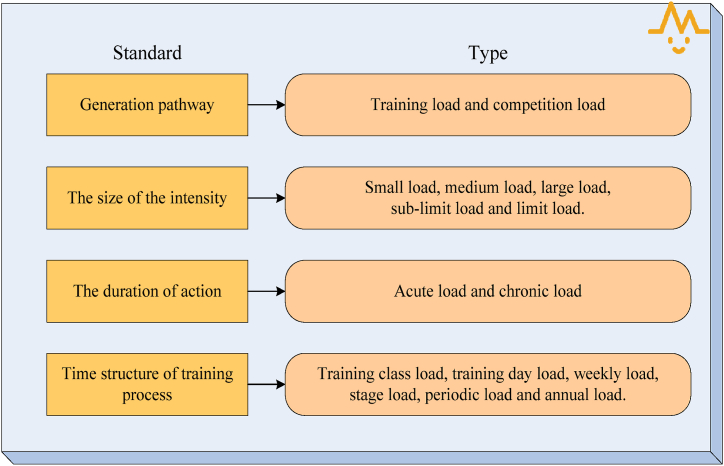
Classification of exercise load according to different standards.

Logically speaking, to clarify the connotation of sports load, there are five views on its essence. First, the exercise load is essentially an incentive, that is, multiple training methods respond positively to the functional system of various parts of the body. Second, the essence of exercise load is a reflection, which is a kind of high-intensity physiological stress or physiological response produced by athletes in a specific sports environment. Third, the essence of exercise load is a kind of pressure, that is, the stress of athletes in a series of sports. Fourth, the exercise load is essentially “stimulus + response”, that is, starting from the training object, the exercise load is the response to the training object. Fifth, the exercise load is essentially a kind of work load. Its manifestation refers to various physiological loads that the body can bear during physical training [20]. There are still many arguments about the nature of exercise load, most of which are workload, stimulation, and reaction. This paper comprehensively considers the connotation and actual needs of sports and points out that the essence of sports load is workload, stimulation is the mode of action of sports load, and reaction is the physiological and psychological impact of sports load on the body. In the field of sports, according to different classification standards, the physical load of athletes can be divided into different types. Among them, physiological and psychological loads are generated after the load, rather than on their own. Therefore, they are divided into internal and external loads. Aerobic, mixed oxygen, anaerobic, physical strength, technology, tactics, psychological ability, intelligence, etc. are all ways of exercise, not simply to load oneself [21,22]. Therefore, after comprehensive consideration of the actual situation of physical exercise and the relevant theoretical knowledge, the classification of the exercise load according to different standards is shown in Fig. 3.

As shown in Fig. 3, according to the method of load generation, it can be divided into training load and competition load. According to the load intensity, it can be divided into small load, medium load, large load, sub-extreme load, and extreme load. Depending on the duration of the load, it can be divided into acute load and chronic load. According to the time structure of the training process, it can be divided into training course load, daily load, weekly load, stage load, periodic load, and annual load. The load of exercise is the central factor of training, which plays a dual role in the bodies of athletes. That is to say, an adequate amount of exercise can improve athletes' physical conditions, improve athletes' sports ability, and allow athletes to obtain good sports results. However, excessive exercise load would cause a series of negative effects, such as fatigue, joint pain, etc., that would reduce the effect of training and even cause sports injuries and diseases. At the same time, a large number of training practices also show that maximum intensity training cannot achieve the best training effect. Therefore, it should be understood according to its relationship with sports performance and sports injury. Proper exercise load can promote sportsmanship and athletic performance of athletes [23]. Genetic factors, lifestyle, training load effect and other factors would have a certain impact on athletes' sports ability, and the training load effect has the greatest impact on them. The results show that the correlation between athlete body load and competitive ability is not a straight line, but an inverted U-shaped curve. That is, the body load of the athletes is too large or too small, which does not have much effect on improving competitive ability. Only when certain biological adaptability is achieved can the body constantly adapt to the new load, thus breaking through the original competitive level, to achieve the goal of balanced development. At present, there are many theories and theoretical models about this problem, and adaptation theory is widely accepted.

**Fig. 4:**
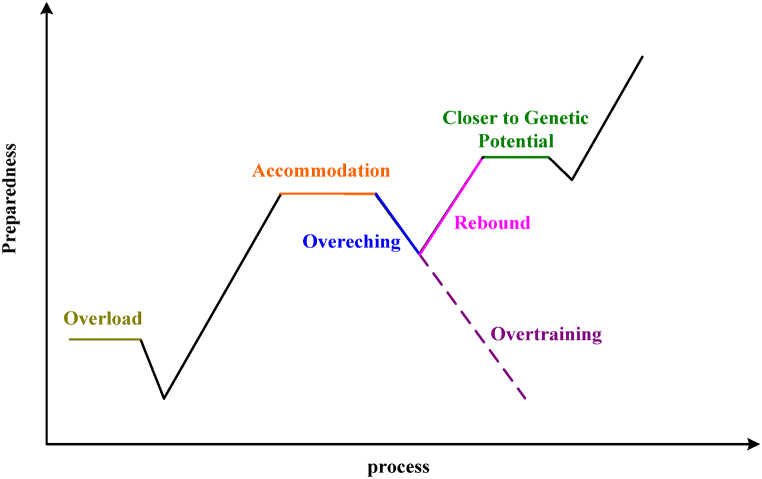
Chart of the sports training adaptation process.

According to adaptation theory, when the body is affected by external stressors, specific or unspecified stress reactions would occur. These stressors are mainly made up of specific diseases and nonspecific diseases, and the nonspecific response of the body to nonspecific diseases is called general adaptation. This theory provides a biological basis for the future practice of physical exercise, which is elaborated in detail by the stress adaptation theory [[24], [25], [26]]. The process of adaptation of exercise training to the theory of adaptation to stress is shown in Fig. 4.

As shown in Fig. 4, the theory of stress adaptation believes that the greatest pressure in sports training comes from various training methods. When the athlete's body is stimulated by different training methods, the body would appear with temporary vigilance, and the functions of various organs of the body would temporarily decrease [27,28]. With continuous exercise, the secretion of hormones would become more and more, and the body functions would gradually become active, which is called adaptation. In long-term exercise, all parts of the body would be exhausted due to physical exertion. If this continues, it is easy to lead to overtraining of the body, which leads to degradation of body functions and various injuries. After a period of rest and adjustment, physical quality would be greatly improved, so it could better adapt to the new environment and improve our physical quality.

Improper exercise load is considered to be one of the risk factors leading to sports injuries and diseases, and its various degrees of injury to athletes' bodies are related to various factors, such as excessive load intensity, rapid changes in load, long-term accumulation of load, etc. The rapid change in acute load, the negative accumulation of chronic load, and the ratio of acute load to chronic load are considered the three most important factors that lead to sports risk. These factors should be taken into account when monitoring sports risk. The correlation between acute load and sports injury manifests itself mainly in a sudden change in acute load. The load that athletes bear in sports training and competition is not static [29,30]. In a specific training stage, the load of athletes would change dramatically. For example, in pre-contest training, athletes need to conduct comprehensive training, and in this process, athletes would have a higher chance of injury. Due to the drastic change of load, the physiological and mechanical characteristics, EMG release, maximum torque angle, etc. of athletes have changed, and the body functions of athletes cannot adapt to the new load, leading to sports injuries of different degrees. The extraordinary competitive level shown by excellent athletes in the competition is by no means overnight, but the accumulation of the training load effect over the years. Long-term load accumulation effect, that is, the athlete's body would change under the influence of the sports load. With time, this change would gradually accumulate to achieve a change from quantity to quality. A good cumulative effect can promote the physical health of athletes; otherwise, it would cause malignant changes and increase the probability of injury. The acute and chronic load ratio describes the relationship between sports injury and load change. When long-term load accounts for a large proportion and acute load accounts for a low proportion, it can be considered a good physical function. On the contrary, if the acute load rate is high and the long-term load rate is low, it should be considered that the body is not fully prepared and the risk of exercise load would increase.

Through monitoring of sports load, coaches and scientists can quickly grasp the adaptation of sports load to formulate the corresponding control measures. According to current training practice, there are two main aspects of monitoring exercise load. One is to use training indicators, such as the number of times, groups, time, intensity, etc. to measure the load. Second, physiological and biochemical indicators such as heart rate, blood lactic acid, subjective physical sensation, and psychological indicators are used to indirectly reflect the body's adaptation to the load. The first is an indicator to measure exercise load and is the basis and premise to measure exercise load, while the latter is an important means to measure human body fitness for load. At the same time, different monitoring indicators have their own advantages and limitations. Therefore, in the implementation process, appropriate monitoring indicators should be selected according to the different needs of the project.

## 3 Construction of a yoga sports load monitoring neural network model based on ACO

### 3.1 ACO neural network

Nature always gives people rich creativity, and people's cognition of things is the interaction with nature. Through long-term observation, biologists have found that the intelligence of ants is not high, which seems to lack a unified command, but they can cooperate, gather food, build beautiful nests, raise the next generation, and use collective power to exert power beyond personal intelligence. The ACO algorithm is a new algorithm based on ant colony search and ant nest. This method has been applied to the study of tourist agency problems, assignment problems and scheduling problems, and a series of good experimental results have been obtained. The ACO algorithm has not been studied for a long time. Its mathematical foundation is not as good as the genetic algorithm and the simulated annealing algorithm, but its application effect is very good. The ACO algorithm is the most popular field at present and has made great progress both in theory and in practical application.

In the ACO algorithm, a random rate is introduced to simulate the random behavior of the ants during the search process. The random rate is usually used to control the probability that the ants will choose the next move, thereby introducing a certain degree of randomness in the search space to prevent the ants from falling into the optimal local solution. In the ACO algorithm, the random rate can be defined as a probability value between 0 and 1, indicating the probability that the ants will choose different paths. The higher the random rate, the greater the randomness of ants choosing different paths and the stronger the exploration in the search process. By adjusting the random rate size, the exploration and utilization behavior of the ants can be balanced, and the global search ability and the convergence speed of the algorithm can be improved.

The ACO algorithm is a heuristic random search algorithm, which is optimized on the basis of simulated annealing, genetic algorithm, tabu search, artificial neural network, and other algorithms. The ACO algorithm shows a strong advantage in finding better solutions. It makes full use of the positive feedback principle, accelerates the evolution process to a certain extent, and is also an essentially parallel algorithm. Through the constant exchange and transmission of information, they can cooperate to find better solutions. The artificial ACO algorithm is a method to find the shortest path by imitating actual ants. This method absorbs some typical characteristics of the ant colony: Finding the situation in a small range to determine whether there is food or similar pheromones. It can release its own hormones. The number of pheromones would gradually decrease.

Ant Colony Optimization (ACO) is a heuristic optimization algorithm inspired by the foraging behavior of ants. Solve combinatorial optimization problems by simulating the behavior of ants releasing pheromones, perceiving the environment, and communicating with each other when looking for food. The ant colony algorithm is used mainly to solve combinatorial optimization problems, such as the travel salesman problem, the assignment problem, and the scheduling problem. Its basic idea is to guide the ants in finding the optimal solution in the solution space through information transmission and pheromone release between the ants in the ant colony. The core idea of the ACO algorithm is the positive feedback principle; that is, when ants explore the solution space, they make decisions based on the pheromone concentration on the existing path and then continuously optimize the search path.

The Ant Colony Optimization (ACO) algorithm is inspired by the behavior of ants in finding food paths. Its features include positive feedback mechanism, jumping out of local optimal solution, adaptive adjustment, etc., making it suitable for various optimization problems and having wide application value.

In this study, the Ant Colony Optimization (ACO) neural network model uses the behavior of ant colonies to simulate the process of finding the optimal solution, thus distinguishing different types of yoga courses and rehabilitation activities to accurately assess the exercise load.

(1) The model extracts and preprocesses the exercise data collected to obtain a data set that describes the characteristics of different activities.
(2) During the neural network training process, the ACO algorithm uses the randomness of the ants in the search space and the transmission of pheromones to optimize the weights, so that the neural network can better distinguish different types of activities and accurately predict the load of the exercise. Through model training and optimization, the neural network can learn patterns and differences between different types of activity, thus achieving an accurate assessment of the exercise load.

This paper introduces the ACO algorithm model and its implementation process with the traveling salesman problem. Let x be the total number of ants in the ant colony. The distance from city m to city n is expressed in $v_{mn}\left(m,n=1,2,\cdots ,y\right)$. If the number of ants in city m at s moment is $v_{m}\left(s\right)$, then there is $x=\sum_{m=1}^{y}v_{m}\left(s\right)$.

The remaining information of branch (m,n) is $\tau_{mn}$ when it is s. At the initial stage, the amount of information on each path is the same, and ant l(l=1,2,⋯,x) would choose their direction of movement according to the number of pheromones on each path. In the basic ACO algorithm, a method called “random ratio” is adopted, which gives the possibility that the ant colony in urban m would migrate to n city, and gives the possibility that the ant l would migrate to m city to n city in s time $q_{mn}^{l}$.

(1) $$q_{mn}^{l}\left(s\right)=\left\{ \begin{array}{c} \frac{{\left[\tau_{mn}\left(s\right)\right]}^{\alpha }{\left[\eta_{mn}\right]}^{\beta }}{\sum_{l}{\left[\tau_{mn}\left(s\right)\right]}^{\alpha }{\left[\eta_{ml}\right]}^{\beta }}\\ 0 \end{array}\right.$$

α is a heuristic factor of information, mn represents the importance of the remaining information on the path; β is the expected incentive factor, which can reflect the relative importance of visibility. In the travel salesman problem, DD is the awareness of the transfer of visibility of prior knowledge to the city.

After the cycle, adjust the information for each route according to (2), (3).

(2) $$\tau_{mn}\left(s+y\right)=\left(1-\rho \right)\cdot \tau_{mn}\left(s\right)+\Delta \tau_{mn}$$

(3) $$\Delta \tau_{mn}=\sum_{l=1}^{x}\Delta \tau_{mn}^{l}$$

Among them, the volatility coefficient of the pheromone is ρ, the residual factor of the pheromone is (1−ρ), and the value of ρ is generally 0–1 to avoid the continuous accumulation of information. $\Delta \tau_{mn}^{l}$ represents the number of pheromones left by the l ant on the (m,n) path in this cycle, which depends on the behavior of the ant. The shorter the path, the more pheromones that would be released. $\Delta \tau_{mn}$ represents the increase of pheromone of (m,n) path in this cycle. According to different algorithms, the expressions of $\Delta \tau_{mn}$、 $\Delta \tau_{mn}^{l}$ and $q_{mn}^{l}\left(s\right)$ are different, which depends on the specific settings of the problem.

The ACO neural network model is an optimization model. The parameters in the ACO neural network model include the number of ants, the fluctuation coefficient of pheromones, the residual factor of pheromones, etc. The number of ants determines the degree of parallelism in the search process, and the fluctuation coefficient and the residual factor of the pheromones affect the update and propagation of the pheromones on the path.

In this experiment, the ACO neural network model is applied to the field of sports load monitoring. Its principle is to use the ACO algorithm to guide the neural network to learn sports training data to predict the training load of athletes. By simulating the behavior of ants when searching for food, the model can better discover the laws and patterns in the data, thereby improving the accuracy and efficiency of prediction.

### 3.2 Design of the load monitoring model for yoga sports

In sports training, it should not only master the intensity of training load, but also understand the physical function of athletes after training. However, there are many factors that affect exercise load. When a single biochemical indicator is used to assess exercise load, there are usually some limitations and deviations. The intensity of exercise was evaluated with blood lactic acid as an index, but it could not accurately reflect the intensity of exercise. At the same time, determination of blood urea cannot accurately reflect the intensity of exercise. Some biochemical indices are related not only to exercise intensity, but also to exercise volume. For example, in high-intensity training, urine excretion would increase. A comprehensive evaluation of blood lactic acid, urine protein, blood urea, and other biochemical indicators showed that blood lactic acid was related to intensity of exercise, while blood urea was related to exercise load. The production of urinary protein is closely related not only to the intensity of exercise, the amount of training, and the functional state of the body.

Theoretically, a feedforward network with a single hidden layer can map all continuous functions, while in the learning process of discontinuous functions, there are only two hidden layers. With increasing number of hidden layers, the non-linear mapping ability of the neural network is improved, but with increasing number of hidden layers, the performance of the network is also affected [31]. This paper uses a three-layer neural network containing one. In neural networks, the number of input neurons is determined by various influence factors [32,33]. The function of the hidden layer node is to extract and store the internal rules of the sample. Each layer node has multiple weights, which is an important indicator of improving network mapping performance. Because the number of nodes in the hidden layer is small, the amount of information obtained from the samples is small, which cannot fully describe the sample rules.

Neural networks improve non-linear mapping capabilities by adding hidden layers, which means adding one or more hidden layers to the network structure. The hidden layer is the layer of the neural network between the input layer and the output layer, which contains the nodes of some hidden units.

Athlete load monitoring technology refers to the technology used to monitor and evaluate the physical load experienced by athletes during training and competition.

The empirical formulas for the hidden layer nodes of the three-layer feedforward network are shown in the following (4), (5), (6):

(4) $$g=\sqrt{t+1}+\alpha$$

(5) $$g=\log\;2^{t}$$

(6) $$g=\sqrt{tp}$$

The number of hidden layer nodes g, the number of input layer nodes t, and the number of output nodes p are all given in the formulas. The value of α does not exceed 10. The number of hidden layer nodes is determined by formula (4).

**Fig. 5:**
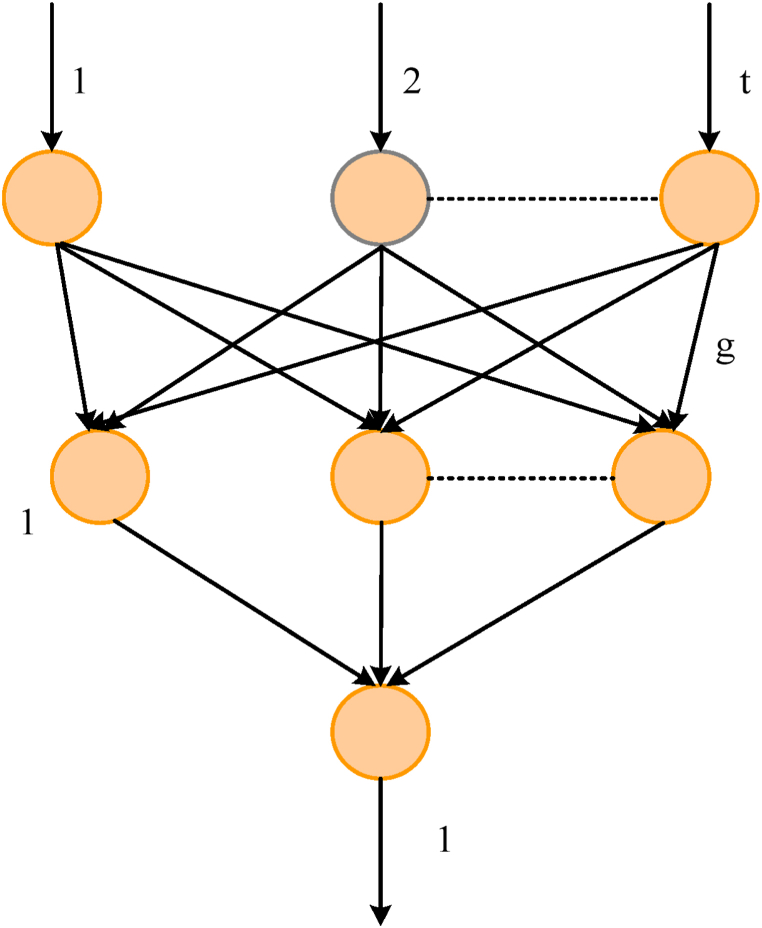
Neural network structure.

The reason why this paper chooses the structure of the ACO-BP neural network is that it combines the traditional error backpropagation (BP) algorithm and the ant colony algorithm. When solving the local minimum and initial value sensitivity problems of the BP algorithm, it uses the global search characteristics of the ant colony algorithm to bring the neural network learning process closer to the optimal solution, thus improving training efficiency and accuracy. Through this combination, the ACO-BP neural network can overcome the limitations of the BP algorithm, better adapt to complex optimization problems, and make network training more robust and reliable. The structure of the neural network is shown in Fig. 5.

As shown in Fig. 5, the most commonly used error backpropagation algorithm (BP) in neural networks is clear in concept, simple in calculation, rigorous in the derivation process, and strong in universality, so it is widely used. However, the essence of this method is local search, which cannot effectively solve the global minima of multiple peak functions, and the convergence time is long, so it is easy to fall into local minima. In this regard, many algorithms have been improved. The simplest and easiest to implement is the variable learning rate BP algorithm, but it is also a local search method and cannot completely avoid falling into a local minimum. On this basis, a feedforward neural network training method based on ACO is proposed. Because the BP algorithm has the disadvantage of having a local minimum, this paper uses the ACO-BP neural network for training. In this method, the neural network learning process can be regarded as an optimal problem, and a group of optimal weight combinations can be obtained to minimize the deviation from the required results. Therefore, the ACO-BP method can effectively solve the problem of local optimization and initial value sensitivity of the BP algorithm by optimizing the initial value of the BP algorithm. On this basis, by introducing the gradient decreasing principle of the BP algorithm, the weight is carefully adjusted to seek the global optimum, in order to effectively solve the quantization error caused by dividing the defined area and the problem that a single ant algorithm spends too much in the network.

It is a difficult challenge to build an exercise load monitoring model of an ant colony optimization (ACO) neural network based on data from yoga training and rehabilitation therapy. Understanding the fundamentals of the ACO algorithm, the neural network's architecture, and how to integrate the two to create a motion load monitoring model in an efficient manner are essential to clarifying the use of the ACO neural network model. To begin with, the ACO algorithm is a bionic evolutionary algorithm that draws inspiration from the biological realm of the natural world. To determine the best course of action, it mimics the way ants locate paths collectively. The ACO algorithm views the ant's walking path as the workable solution to the problem that has to be solved, and the problem's solution space is made up of all the ant population's paths. More pheromones will be released by ants with shorter pathways. The pheromone concentration on the shorter path increases with time and all ants that select this way eventually become more numerous. The optimal solution to the problem to be optimized at this point is the one that the entire ant colony will ultimately focus on under the influence of positive feedback.

We then have to think about how to create a neural network model. A type of nonlinear dynamical system with significant information processing capacity is the neural network. One of the most representative neural network models is the BP neural network. It trains network weights and thresholds using error backpropagation learning methods. BP neural networks do, however, have certain drawbacks, including low efficiency, sluggish convergence, and a propensity to enter local minima. As a result, you might think about optimizing the BP neural network's training procedure using the ACO algorithm. The fundamental concept behind building the ACO-BP neural network model, which combines the ACO and neural networks, is to finish the iterative adjustment of the neural network weight threshold by substituting the ACO algorithm for the conventional BP algorithm. In particular, the neural network's weights and thresholds may be thought of as parameters that need to be optimized. The global optimization features of the ACO algorithm can be utilized to determine ideal weights and thresholds. In this method, the prediction accuracy and generalization capacity of neural networks can be enhanced, as well as the drawbacks of BP neural networks can be avoided.

The ACO-BP method solves the main problems of local optimization and initial value sensitivity of the BP algorithm by optimizing the initial value of the BP algorithm and adopts the idea of the ant colony optimization algorithm. Specifically, the ACO-BP algorithm first uses the ant colony optimization algorithm to optimize the initial weights and biases of the BP neural network, so that the initial state of the neural network is closer to the global optimal solution. The ant colony optimization algorithm is a heuristic algorithm that simulates the behavior of ants looking for food and has global search and adaptability capabilities. When the ant colony optimization algorithm is introduced, the ACO-BP algorithm can effectively find the optimal solution throughout the search space, thus avoiding the dilemma of the BP algorithm falling into the local optimal solution. In the ACO-BP algorithm, the ant colony optimization algorithm continuously adjusts the initial weights and biases of the BP neural network through an iterative search process, so that it gradually tends to the optimal global solution. By optimizing the initial value of the BP algorithm, the ACO-BP algorithm can converge to a better solution faster and has better robustness and stability.

Release all x ants. Ant l selects an element in the set $M_{q_{m}}\left(1\leq m\leq x\right)$ according to the following probability formula.

(7) $$q_{l}\left(m\right)=\frac{\tau \left(m\right)}{\sum_{1\leq n\leq x}\tau \left(n\right)}$$

Record the position where the ants pass through and set a value for this weight. The ants go through after choosing all the weight parameters, and each value is a network parameter. Repeat this procedure until all ants reach their destination. When weighting the weights selected by different ants, the output results and errors are obtained, and the pheromone is updated according to the pheromone adjustment rules.

According to the regulation law of pheromones, the pheromones left over from the past would disappear with the passage of time, and each route needs to be adjusted. Each route must be modified after y time units.

(8) $$\tau_{n}\left(M_{q_{m}}\right)\left(s+x\right)=\rho \tau_{n}\left(M_{q_{m}}\right)\left(s\right)+\Delta \tau_{n}\left(M_{q_{m}}\right)$$

(9) $$\Delta \tau_{n}\left(M_{q_{m}}\right)=\sum_{l=1}^{y}\Delta \tau_{n}^{l}\left(M_{q_{m}}\right)$$

**Fig. 6:**
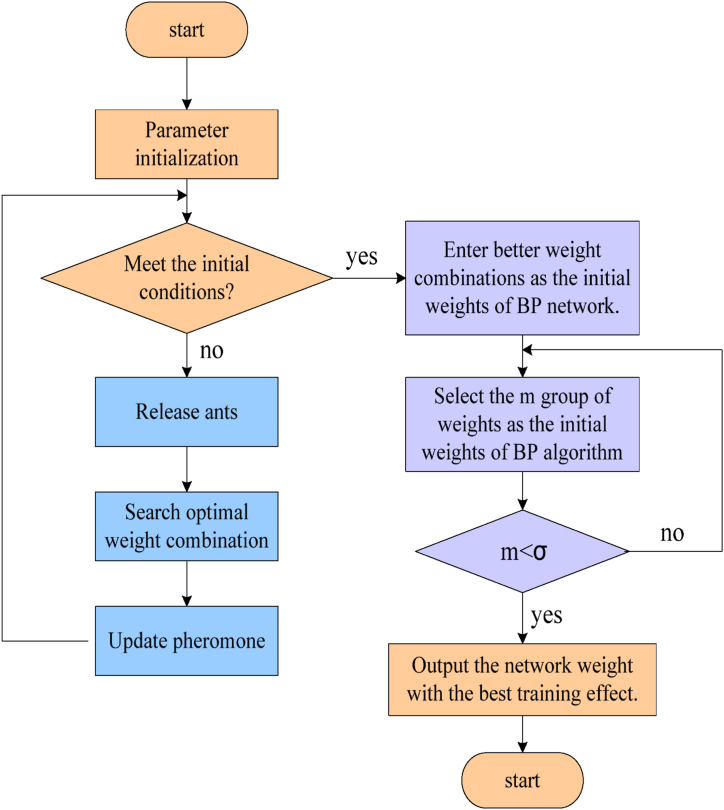
The ACO-BP algorithm process.

$\Delta \tau_{n}^{l}\left(M_{q_{m}}\right)$ represents the pheromone left by the l th ant on the n th element $q_{n}\left(M_{q_{m}}\right)$ of the $M_{q_{m}}$ set in this cycle. In formula (8), s represents the residual factor of pheromone. On the basis of this, the neural network is subsequently trained using the BP method. The process is shown in Fig. 6.

As shown in Fig. 6, it analyzes the error between the network output and the real output, transmits the error through the output layer to the input layer, and adjusts the weight value in the training process. Test the capacity of the neural network for generalization using verification samples. If the error in the result meets the standard, the program would be ended; otherwise, it should start from the first step.

In this study, the ACO-BP algorithm identifies the exercise load by combining ant colony optimization and backpropagation algorithms. First, the physiological and exercise data of the athletes, including heart rate, blood pressure, and other indicators, are collected and pre-processed and input into the ACO-BP neural network model for training. After training, the model can predict new exercise data and output the corresponding exercise load level, helping coaches and athletes understand their current physical state and exercise load level. By evaluating and optimizing the performance of the model, its accuracy and practicality can be continuously improved, providing more targeted guidance and suggestions for sports training.

Furthermore, the neural network model in this article monitors and analyzes the athlete's exercise load using the ACO-BP algorithm, providing a specific way to formulate personalized exercise therapy. The model uses the ant colony optimization algorithm to optimize the initial weights and biases of the BP neural network, improving the efficiency and performance of the training. By timely monitoring and adjusting the exercise load, the athlete's training effect can be maximized, rehabilitation and physical recovery can be promoted, and personalized exercise therapy can be formulated and implemented.

## 4 Experimental analysis of a motor load monitoring model based on ACO neural network

In this experiment, the preprocessing steps are as follows:

(1) Data cleaning: For monitoring data, missing values are first processed. If there are missing values in the monitoring data, the interpolation method is used to fill in the samples where the missing values are located. In addition, this experiment uses visualization methods to detect outliers.
(2) Normalization of the data: In this experiment, different indicators or features have different measurement units and scales. To ensure the robustness of the model, Min-Max normalization is used to normalize the data.
(3) Feature selection: To select the most relevant feature subset to improve the performance and generalizability of the model, in this experiment the principal component analysis (PCA) method is used to evaluate the importance of features and select the best feature subset.
(4) Data transformation: In this experiment, some features need to be transformed to make them more suitable for the model algorithm. For skewed distributed data, a logarithmic transformation is applied to make it more symmetric to meet the assumptions of the model.

### 4.1 Experimental results of the ACO neural network algorithm

To verify this network model, a computer simulation test must be carried out. Taking blood indicators of female athletes as an example, this article used the National Basketball Association information platform to sample 154 monitoring data, due to the small number of data sets and in order to ensure the speed of operation, and selected 140 as training samples and 14 as test samples. In this paper, the ant colony size x=30 is selected, and the domain is divided into 50 equally.

The data set used in this paper covers the blood indicators of female athletes and is obtained by sampling the monitoring data from the National Basketball Association Information Platform. The specific sample selection process involves the following steps:

(1) Sample sampling: 154 monitoring data from the National Basketball Association Information Platform were selected as a dataset, covering the blood indicators of female athletes over different time periods. (2) Sample segmentation: The 154 monitoring data were divided into two parts, 140 of which were used as training samples and the remaining 14 as test samples.

During the sample selection process, it is necessary to exclude abnormal samples and handle missing samples. First, exclude samples with abnormal blood indicators and other abnormalities. Second, the interpolation method is used to supplement the samples of important blood index data.

In this experiment, the division strategy of dividing the data set into training samples and test samples is of key significance. First, it helps prevent the occurrence of overfitting problems and improves the generalizability and reliability of the model in practical applications. Second, by comparing the performance of different models and combinations of parameters in the test set, select the best model and parameters to improve the overall performance of the model.

However, the data set splitting strategy also has some potential limitations. First, if the data distribution between the training set and the test set is uneven, it can affect the accurate evaluation of the model performance. Second, there is a risk of information leakage. If the test set is used to guide model training or parameter selection during the model selection and tuning process, the final performance evaluated may be overly optimistic.

In Table 1, the parameter settings are displayed.

**Table 1:** Parameter settings of the ACO-BP algorithm and the BP algorithm.

Name	ACO-BP	BP
Ant colony size(x)	30	–
Minimum value of weight interval	−3	−0.2
Maximum value of weight interval	3	0.2
Domain of definition	50	–
Y_BP_	13000	19000
R_0_	0.004	0.004

As shown in Table 1, Table 3, it indicates that athletes are in poor physical condition and cannot adapt to the current training program, so the amount of training should be adjusted in time. It can use 0 to indicate that the athlete can adapt to the training program at that time and can continue to implement the training program if the amount of exercise is appropriate. The maximum value of the weight interval in the experiment is 3, which means that the physical function of the athletes has reached a completely normal level and the effect of training can be improved by increasing the amount of training. Table 2 shows the test results of the simulation experiment.

**Table 2:** Simulation test results.

Method	Mean square error		
	I = 200	I = 600	I = 800
Standard BP algorithm	0.008663	0.005231	0.003187
BP algorithm with momentum term	0.007571	0.003052	0.0005725
ACO-BP algorithm	0.006013	0.001879	7.8316e-4

**Table 3:** Data results after neural network training.

Sample sequence number	Output sample	Forecast error
1 sample	0.954	0.022
2 sample	0.416	−0.053
3 sample	0.422	−0.023
4 sample	0.11	0.124
5 sample	0.373	−0.099
6 sample	0.292	0.016
7 sample	0.276	0.003
8 sample	0.283	0.021
9 sample	0.063	−0.012
10 sample	0.027	−0.027
11 sample	0.331	0.025
12 sample	0.104	0.061

As shown in Table 2, the experiment shows that the ACO-BP algorithm outperforms both the regular BP algorithm and the enhanced BP algorithm with the momentum term in terms of convergence speed and average error. It shows that the algorithm has greater advantages and more stable advantages. In view of the defects of the single ACO algorithm and the BP algorithm, the ACO-BP algorithm proves that the ACO neural network has high monitoring accuracy and fast prediction speed and is superior to the BP neural network model. This algorithm can effectively detect sports load.

### 4.2 Results of the model and reliability test based on yoga training data

To test the results and reliability of the model, experiments were conducted on the basis of actual yoga training data. The subjects of this experiment were 30 sports majors from a sports college, 15 boys, and 15 girls. Among them, there are two international athletes, 12 national athletes, and 16 national level athletes. Through research on athletes, it is found that after completing yoga training, the athletes have performed a series of relaxation exercises. Compared to traditional relaxation exercises, they can significantly reduce the concentration of lactic acid after training and reduce the physical fatigue of sports. In addition, it can also relieve jump acceleration in the high-strength training center and reduce body fatigue. Therefore, the data generated during their yoga training are combined with the model to calculate the sports loading monitoring data.

**Fig. 7:**
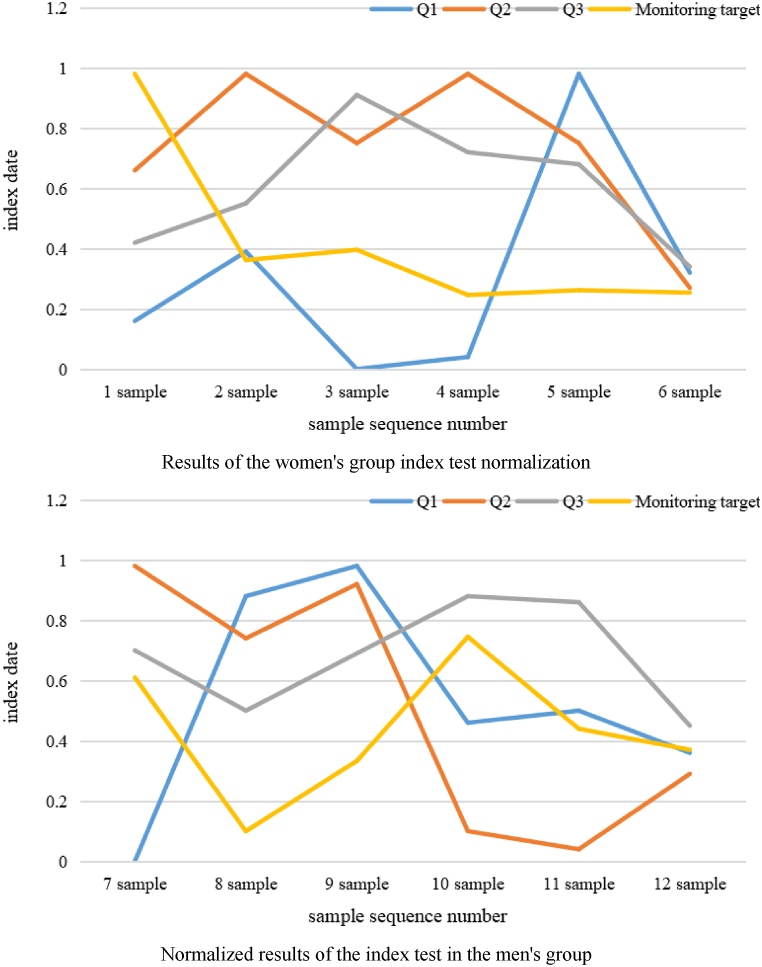
Index test normalization results.

Through a large number of training and prediction tests, it is found that the increase in some test data would have a certain impact on the convergence and prediction accuracy of the model, such as athletes who are not experienced in training or because of some specific circumstances that have bias, so such data would be removed, and the final normalization results are shown in Fig. 7.

As shown in Fig. 7, the selected indicators and monitoring data are standardized and used as training samples for the ACO neural network. On this basis, the improved algorithm is used to process the training samples. The traditional BP algorithm uses the maximum velocity gradient method to correct for weight. This method gradually approximates the minimum point from the initial point to the tilt direction of the error function, so it is 0. Because the gradient decline method has a fast decline speed in the first few stages, but when approaching the optimal value, because the gradient change trend tends to be consistent, the decay of the error function becomes very slow. The data of the input layer and the output layer obtained after ACO neural network training are shown in Table 3.

**Fig. 8:**
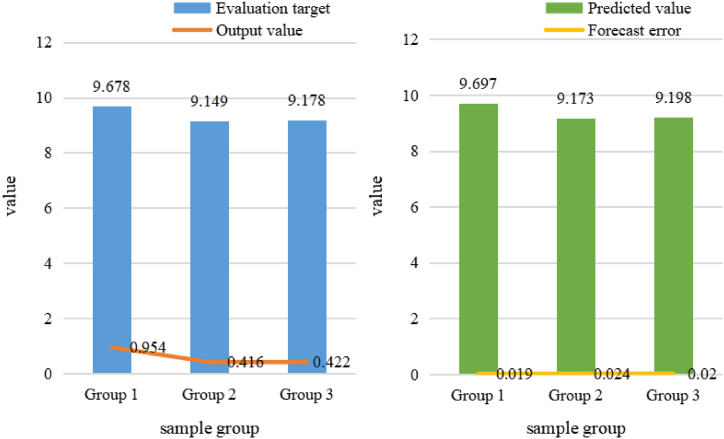
Fitting precision value of prediction model.

As shown in Table 3, the results show that the ACO neural network model has high prediction precision with an error range of 0.1 to monitor the exercise load based on yoga training data. The model is accurate and effective. Then, the ACO neural network method is used to predict the three sample groups and compare them with the measured values to obtain the fit accuracy of the model, as shown in Fig. 8.

As shown in Fig. 8 (a), the evaluation objectives of the three data groups are in the range of 9.1–9.7, and the model output values are 0.954, 0.416 and 0.422, respectively. There is no significant difference among the three groups of sample data. As shown in Fig. 8 (b), the range of the three groups of prediction values is also between 9.1 and 9.7. The prediction errors calculated from the three groups of data are 0.019, 0.024 and 0.02, respectively. All prediction error values are within 0.1, indicating that the model is in good shape.

Data are input after normalized processing according to the standard, and the trained ACO neural network is called with the function. Based on data from yoga training based on athletes, the actual data of exercise load detection is obtained through inverse normalization processing. The results of the specific data are shown in Table 4.

**Table 4:** Model accuracy results.

Real Data		Q_test
Actual value		9.109
Neural Network Model	Predicted Value	9.106
	Error Value	−0.003

As shown in Table 4, the predicted value is 9.106. Compared to the actual detection value of 9.109, the prediction error of the constructed ACO neural network model is very small, only −0.003. It shows that the ACO neural network model can well fit the functional relationship between yoga training level and exercise load, and has high prediction accuracy. According to the above experiments, it is suggested that coaches and athletes pay attention to the value and function of yoga relaxation exercise, and pay attention to the role of heart rate, subjective exercise level, and mood in evaluating and monitoring exercise load. Attention should be paid to meditation, breathing, and exercise in yoga, and posture of all parts of the body during exercise to reduce exercise fatigue.

### 4.3 Significance test

For the parameters of the algorithm in Table 1, a significance test is now performed. The specific steps are as follows.

First, assume that these parameters have no significant impact on the output results of the neural network model, then select an appropriate significance level of 0.05 to represent an acceptable error probability level, run the neural network model using the parameters given, and record the output results. Finally, statistical tests are performed using appropriate t tests for each parameter to test the hypotheses. On the basis of the results of the statistical test, determine whether each parameter has a significant impact on the model results. If the p-value is less than the selected significance level, reject the null hypothesis, indicating that the parameters have a significant impact on the model results. The results of the significance test are shown in Table 5.

**Table 5:** Significant empirical results.

Parameter	p value	Significance level	Completion
Ant colony size(x)	0.023	0.05	Significantly
Minimum value of weight interval	0.135	0.05	Not obvious
Maximum value of weight interval	0.002	0.05	Significantly
Domain of definition	0.087	0.05	Not obvious
Y_BP_	0.001	0.05	Significantly
R_0_	0.072	0.05	Not obvious

As can be seen in Table 5, the size of the ant colony and the maximum weight interval, YBP, have a significant impact on the model results, while the minimum weight interval, the definition domain, and R0 have no significant impact on the model results.

## 5 Experimental discussion

The ACO neural network model has good prospects for development in exercise load monitoring and can be adjusted and extended to be applied to other training methods.

First, verify the applicability of the model in different sports or types of sports.

Second, the ACO neural network model can be combined with other training methods to explore its application effects in different training projects.

The ACO neural network model is of important practical importance in real-life sports training and performance monitoring environments.

(1) The ACO neural network model provides personalized training guidance for athletes, tailors appropriate training plans based on the physical condition of the athletes, training goals, and personal characteristics to improve training pertinence and effectiveness.
(2) The ACO neural network model can provide new methods and tools for sports scientific research, help researchers gain a deeper understanding of the impact of exercise load on athlete physical functions and sports performance, and promote the development of sports training and sports.

## 6 Conclusions

With the increasing amount of data in the Internet of Things, utilizing data mining techniques to extract potential knowledge or patterns from data such as images generated by the Internet of Things will enable the Internet of Things to provide intelligent services. At present, data mining algorithms require a large amount of computation to classify image data, so it is necessary to transfer data from IoT application scenarios such as smart homes to cloud computing servers for processing. Machine learning is an efficient algorithm, so one of the relatively mature and stable neural network algorithms is selected for experimental analysis in the motion training data set. At present, the sports industry has developed a set of scientific physiological and biochemical indicators for athletes. It can scientifically monitor and analyze the sport function and make it standardized, standardized, and systematic. However, the coaches' quantitative analysis of the athletes' comprehensive indicators and the training plan based on the quantitative body load are not very complete. However, yoga training can alleviate physical fatigue in athletes to some extent, restore physical functions, and help athletes recover from sports injuries. This article uses the ACO neural network model to monitor and analyze athletes' exercise load based on the yoga training data of athletes, which is conducive for coaches to regulate training and competition training programs. It can improve the quality and level of training, provide scientific and effective guidance for athletes' sports training, so as to improve athletes' sports performance. Through the comprehensive analysis of various athlete indicators, a sports load prediction model based on the ACO neural network is established. The predicted value is basically consistent with the measured value, achieving the expected purpose, which is conducive to guiding coaches to conduct sports training scientifically. However, this article is only a preliminary study, which needs to be further improved due to technology and ability limitations. In the future, other machine learning algorithms and high-performance computing would be further optimized.

This study demonstrates the potential of the ACO neural network model in monitoring exercise load, but there are still some limitations. There is subjectivity and uncertainty in the parameter selection and adjustment process in the model, and more experiments and verification are needed to determine the optimal parameter settings. Furthermore, this study did not take into account other factors that may affect exercise load, such as climate and environment, so these factors must be considered in further detail in practical applications.

Future research can further improve and expand this study in the following areas.

(1) Expand the sample size and verify it under different groups and environmental conditions to enhance the generalizability of the model.
(2) Explore the application of other machine learning algorithms and deep learning models in exercise load monitoring to improve the accuracy and stability of the prediction of the model.
(3) Further optimize the model parameter selection and adjustment process, adopt automated and intelligent methods to improve model performance and efficiency, better utilize the ACO neural network model to monitor and evaluate exercise load, and provide more scientific and effective guidance for sports training.
(4) Pay more attention to the impact of yoga on athletes' metabolism. Through rigorous experimental design and monitoring of physiological parameters, we will deeply explore the role of yoga in the regulation of athletes' body metabolism and provide a scientific basis to optimize athlete training and competitive performance.

## Data availability statement

Data will be made available on request.

## CRediT authorship contribution statement

**Wenhui Ma:** Writing – original draft, Visualization, Validation, Supervision, Software. **Bin Guo:** Writing – review & editing, Software, Formal analysis, Data curation, Conceptualization.

## Declaration of competing interest

The authors declare that they have no known competing financial interests or personal relationships that could have appeared to influence the work reported in this paper.
